# Resveratrol activates autophagy and protects from UVA-induced photoaging in human skin fibroblasts and the skin of male mice by regulating the AMPK pathway

**DOI:** 10.1007/s10522-024-10099-6

**Published:** 2024-04-09

**Authors:** Yangmin Xia, Hao Zhang, Xiangyi Wu, Ye Xu, Qian Tan

**Affiliations:** Department of Burns and Plastic Surgery, Nanjing Drum Tower Hospital, Affiliated Hospital of Nanjing University Medical School, Nanjing, China

**Keywords:** Resveratrol, Autophagy, AMPK, UVA, Photoaging, ROS

## Abstract

Skin photoaging is mostly caused by ultraviolet A (UVA), although active medications to effectively counteract UVA-induced photoaging have not yet been created. Resveratrol, a naturally occurring polyphenol found in the skin of grapes, has been shown to have various biological functions such as anti-inflammatory and antioxidant characteristics. However, the role of resveratrol in UVA-induced photoaging has not been clarified. We investigated the mechanism of action of resveratrol by UVA irradiation of human skin fibroblasts (HSF) and innovatively modified a mouse model of photoaging. The results demonstrated that resveratrol promoted AMP-activated protein kinase (AMPK) phosphorylation to activate autophagy, reduce reactive oxygen species (ROS) production, inhibit apoptosis, and restore normal cell cycle to alleviate UVA-induced photoaging. In addition, subcutaneous injection of resveratrol not only improved the symptoms of roughness, erythema, and increased wrinkles in the skin of UVA photodamaged mice, but also alleviated epidermal hyperkeratosis and hyperpigmentation, reduced inflammatory responses, and inhibited collagen fiber degradation. In conclusion, our studies proved that resveratrol can treat UVA-induced photoaging and elucidated the possible molecular mechanisms involved, providing a new therapeutic strategy for future anti-aging.

**Supplementary Information:**

The online version contains supplementary material available at 10.1007/s10522-024-10099-6.

## Introduction

The most prevalent manifestation of aging is skin aging, which has histologic characteristics like collagen degeneration and decreased extracellular matrix (ECM) (Lee et al. 2021) as well as clinical symptoms including increased wrinkles, hyperpigmentation, and skin slackening (Rittie and Fisher 2015).In addition to having an aesthetic impact, photoaging is intimately linked to diseases like seborrheic keratosis, age spots, skin cancer, and other illnesses (Rabe et al. 2006). Long-term ultraviolet (UV) exposure is the most important cause of photoaging (Gilchrest 2013).

UV is mainly divided into short-wave ultraviolet (UVC, 100–280 nm), medium-wave ultraviolet (UVB, 280–320 nm), and long-wave ultraviolet (UVA, 320–400 nm) (D’Orazio et al. 2013). UVC is almost completely absorbed by the ozone layer, with UVA (95%) and UVB (5%) reaching the ground. UVB is mainly absorbed by the epidermis, whereas UVA is not only absorbed by the epidermis but also reaches deeper into the dermis (Salminen et al. 2022), which is the main cause of photoaging (Battie et al. 2014). Long-term UVA exposure inhibits skin fibroblast autophagy (Jeong et al. 2020). It generates excessive ROS (Amaro-Ortiz et al. 2014; Chen et al. 2021), as well as causing the secretion of various inflammatory factors and a large amount of matrix metalloproteinase (MMP), which accelerates the breakdown of collagen fibers and ECM, increasing skin wrinkles and hyperpigmentation (Amano 2016; Rabe et al. 2006; Rittie and Fisher 2015). Autophagy is a cellular self-preservation and degradation process that maintains the balance of the internal and exterior cellular environment by eliminating certain aging or damaged organelles and macromolecular proteins (Kelekar 2005; Wollert 2019). Research has demonstrated that autophagy is crucial in slowing the aging process (Salminen and Kaarniranta 2009), particularly in regulating skin aging (Eckhart et al. 2019). Therefore, promoting autophagy may be a potential strategy for the prevention and treatment of UVA-induced photoaging.

AMP-activated protein kinase (AMPK) is an important pathway in the regulation of autophagy (Meijer and Codogno 2007; Mihaylova and Shaw 2011), which can activate autophagy-initiating proteins (Unc-51 like autophagy activating kinase, ULK1) directly or by inhibiting the Mechanistic target of rapamycin (mTOR) (Cao et al. 2021; Kelekar 2005; Lapierre et al. 2015; Meijer and Codogno 2007; Wollert 2019). Resveratrol is a naturally occurring autophagy regulator that is found in blueberries (Pallauf and Rimbach 2013), grape skins, and other foods (Pastor et al. 2019). It can activate autophagy through AMPK for the prevention and treatment of a number of diseases (Burkewitz et al. 2014; Pyo et al. 2020; Singh et al. 2019), such as cardiovascular disease (H. Li et al. 2022a, b; Song et al. 2018), neuropathy (Han et al. 2014; Yang et al. 2016), and cancer (J. Li et al. 2022a, b; Puissant and Auberger 2010). However, whether resveratrol can activate autophagy to ameliorate UVA-induced photoaging via the AMPK signaling pathway has not been reported.

In this study, we creatively enhanced the photoaging mouse model based on the research of Liang et al. (LH 1989 Sep; Liang et al. 2007; Wirnitzer et al. 2006). This offers a new therapeutic approach for future anti-aging by examining the effects of UVA irradiation on mouse skin and human dermal fibroblasts, as well as looking into the part that resveratrol administration played in the photoaging model to reveal the molecular mechanism of resveratrol’s anti-aging effects.

## Materials and methods

### Antibodies and regents

Autophagy inhibitor 3-methyladenine (3-MA) was purchased from Absin (Shanghai, China); AMPK inhibitor Compound C (CC) was purchased from Abcam (Cambridge, MA, USA); Fetal bovine serum (FBS) was purchased from ExCell Bio (Suzhou, China); DMEM high glucose, trypticase, Resveratrol, PBS, Reactive oxygen species assay kit, Ultrasensitive ECL chemiluminescence kit, Cellular senescence β-galactosidase staining kit, Cell cycle assay kit, BCA protein concentration assay kit, HRP-labeled Goat Anti-Rabbit IgG (H + L) (1:1000, A0208) were from Beyotime (Shanghai, China); 8-methoxypsoralen (8-MOP) from Aladdin (Shanghai, China); RIPA lysate from Solarbio (Beijing, China); Protein Marker, PAGE Gel Rapid Preparation Kit 12.5% from EpiZyme (Shanghai, China); Apoptosis Detection Kit was purchased from Novozymes (Nanjing, China); Beclin-1 (1:1000, 3495, Cell Signaling Technology: CST, Danvers, MA, USA), p-AMPK (1:1000, 2,535, CST), Collagen I (1:1000, 72026, CST), LC3B (1:1000, CY5,992, Abways, Shanghai, China), AMPK (1:2000, 10,929–2-AP, Proteintech, Wuhan, China), MMP1 (1:1000, 10371-2-AP, Proteintech), p21 (1:1000, 10,355–1-AP, Proteintech), p62 (1:1000, 18,420–1-AP, Proteintech), Fluorescenin (FITC)-conjugated Affinipure Goat Anti-Rabbit lgG(H + L) (1:5000, SA00003-2, Proteintech).

### Animals

Six-week-old BALB/c male mice (18–22 g) were housed in cages with bedding, controlled temperature (23 ± 2 °C), humidity (50 ± 5%), and illumination (12 h light/dark cycle). Mice were acclimatized to the facility for 1 week before the experiment. They were divided into a blank group of 6 and a UVA group of 24 (6 mice in each group of UVA, UVA + RSV, UVA + 3-MA, and UVA + PBS). The mice were first placed in a gas anesthesia machine, and their back hair was removed when they were anesthetized. Mice in the UVA group were coated on their backs with 8-MOP and placed in the UV weathering test machine (Suzhou, China). Then, we gave a minimal erythema dose of 0.35 J/cm^2^ UVA, and the dose was increased by 5% per day for 8 weeks of modeling. Then PBS, 3-MA, and 100 μmol/L RSV were given from the 9th week onward and administered two times a week for 8 weeks (Hecker et al. 2021; Zhang et al. 2021). All animal experiments were approved by the Animal Investigation Ethics Committee of Nanjing Drum Tower Hospital (Approval No. 2022AE01020) and conducted following ethical norms.

### Cell culture

The human skin fibroblasts (HSF) purchased from Procell Life Science & Technology Co., Ltd. (Wuhan, China), were resuscitated and cultured in DMEM containing 10% FBS at 37 °C in an incubator with 5% CO_2_.

### Cell viability analysis

Add an appropriate amount of resveratrol to PBS to configure the concentration we need. HSF was digested and centrifuged, the cells were resuspended by adding medium, evenly spread in 96-well plates, incubated overnight in the incubator, and then 4, 8, 12, 16, 20, 24, 28 and 32 J/cm^2^ UVA were given after evenly spreading the plates and incubating overnight, 5, 10, 25, 50, 75, 100 and 200 μmol/L resveratrol (Hecker et al. 2021) was added and incubated for 6 h, and 2 replicate wells were set up, and the plates were incubated for 2 h. Then 16 J/cm^2^UVA was given, and after modeling, the plates were put into the incubator overnight, then 10 μl CCK-8 solution was added to each well (taking care to avoid air bubbles), and the incubation was continued for 2–3 h in the incubator, and the absorbance value at 450 nm was measured in the enzyme marker.

### Annexin V-FITC/PI staining

Cells were spread onto 12-well plates and grouped as indicated for the control, UVA, UVA + 3-MA, and UVA + RSV. A total of 12 sterile centrifuge tubes were labeled with different dyes, including dye-free, FITC single-positive, PI single-positive, control double-dye, UVA double-dye, UVA + 3-MA double-dye, and UVA + RSV double-dye. By digestion and centrifugation, cells from several groups—including adherent and supernatant suspension cells—were gathered. PBS solution was added to the cell sediment in each group, and the cells were then centrifuged once more after being repeatedly gently blown over. Each group of centrifuge tube cell sediment should have 100 μl of Binding Buffer solution added before resuspending. According to the marking on the wall of each centrifuge tube, add 5 μl of dye and incubate for 15 min at room temperature, avoiding light. 400 μl of Buffer solution was added again, and the cells were detected by flow cytometry within 1 h.

### Oxidative stress assay

Cells were inoculated in 24-well plates, divided into Control, Control + 100 μM RSV, Control + 3-MA, UVA, UVA + 3-MA, UVA + 10 μM RSV, UVA + 50 μM RSV, UVA + 100 μM RSV, and after modeling, the DCFH-DA was diluted according to the serum-free culture solution of 1:1000 to make the final concentration of 10 μmol/L. Cells were deprived of the original culture medium, and the prepared working solution was incubated at 37 °C incubator for 20 min, then washed three times with serum-free culture medium, and then observed under a fluorescence microscope and pictures. Finally, the ROS fluorescence intensity was analyzed by ImageJ.

#### Senescence-associated β-galactosidase (SA-β-gal) staining

Cells were inoculated in 12-well plates, and divided into Control, UVA, UVA + 3-MA, and UVA + RSV groups, after modeling, the original culture solution was aspirated, washed with PBS, and aspirated, 1 mL of β-galactosidase staining fixative was added, and the cells were fixed for 15 min at room temperature. After aspirating the fixative and washing with PBS 3 times, 3 min each time, finally, add the staining working solution according to the instructions, seal the 12-well plate with parafilm to prevent evaporation, incubate overnight at 37 °C, and then observe under the optical microscope. The percentage of SA-β-gal positive cells was determined with ImageJ.

### PI staining for cell cycle analysis

Cells were inoculated in 12-well plates and divided into Control, UVA, UVA + 3-MA, and UVA + RSV groups for modeling treatment. Cells were collected into EP tubes by digestion and centrifugation and labeled. The cells were resuspended by adding 1 mL of pre-cooled PBS and centrifuged again to collect the cells, then 1 mL of pre-cooled 70% ethanol was added, blown, mixed, and fixed at 4 °C overnight. The next day, centrifuge and collect the cells, resuspend with PBS, and centrifuge again to collect the cells then add propidium iodide staining working solution, blow to mix the cells, and then 37 °C to avoid the light warm bath for 30 min and complete the flow-through assay on the same day.

### Western blot and immunofluorescence

The samples were lysed on ice with RIPA lysis buffer for 1 h and then centrifuged at 12,000 ×g for 20 min at 4 °C. The supernatant was obtained and stored at − 20 °C. Protein concentration was determined using a BCA protein assay kit. The samples were added to 5 × Sampling Buffer and boiled for 8 min for denaturation. Protein samples were separated on a 12.5% sulfate–polyacrylamide gel (SDS-PAGE) and transferred to a polyvinylidene difluoride (PVDF) membrane. The membranes were closed in TBST-dispensed 5% skimmed milk powder for 2 h at room temperature, and then incubated overnight in the refrigerator at 4 °C with the addition of the corresponding primary antibody. The membrane was washed three times with TBST and then incubated with horseradish peroxidase-conjugated secondary antibody for 1 h at room temperature, before being washed three times with TBST, and finally, the protein blot was detected using an ultrasensitive ECL chemiluminescence kit.

Cells were fixed with 4% paraformaldehyde (PFA) for 20 min, permeabilized with 0.4% Triton X-100 in PBS for 20 min, and then closed for 1 h at room temperature. After closure, cells were incubated with primary antibody LC3B (1:100) overnight at 4 °C, rinsed with PBS, and then incubated with the corresponding secondary antibody for 1 h at room temperature protected from light. DAPI was used for nuclear DNA staining. And after 5 min, PBS was used to wash 3 times. Images were obtained with a Leica SP 8 confocal microscope.

### Histologic analysis

Mice dorsal skin tissues were fixed in paraformaldehyde (4%) for 48 h, decalcified in 15% EDTA for 2 weeks at room temperature, and paraffin-embedded. Sections were then cut, followed by Hematoxylin–Eosin (HE) staining and Masson staining. Photographs were taken for observation using a light microscope and analyzed with ImageJ.

### Statistical analysis

Data were analyzed by GraphPad Prism 9.0 software (San Diego, CA, USA). All data are provided as mean ± standard deviation (SD). The t-test was taken for the results between the two groups. Results above two groups were analyzed by one-way ANOVA or two-way ANOVA with Tukey’s multiple comparison tests. *P* < 0.05 was considered statistically significant.

## Results

### Effect of resveratrol on UVA-induced photoaging

**Fig. 1:**
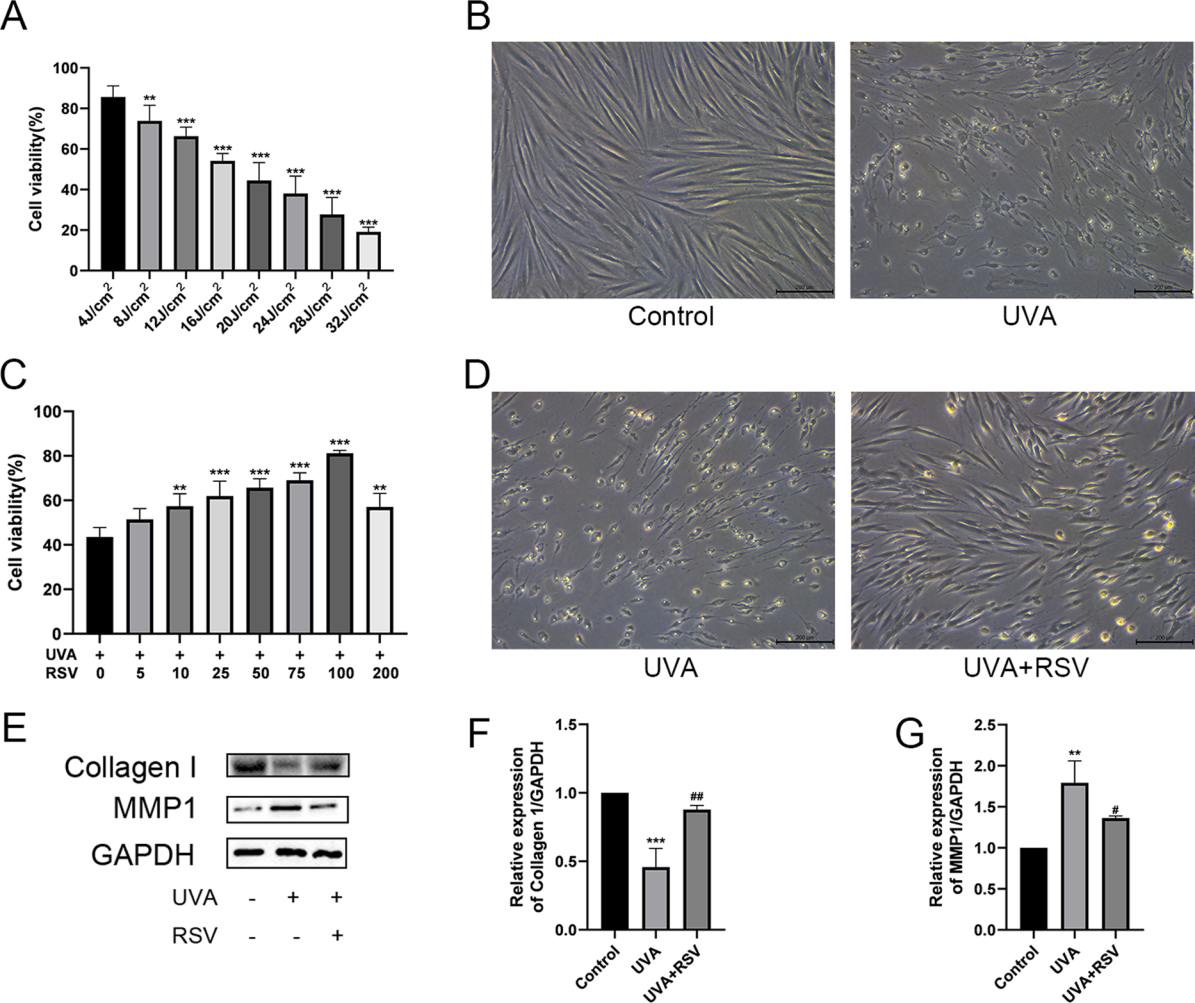
Effect of resveratrol on photoaging due to UVA. **A** HSF cell viability after UVA exposure at different doses, n = 4, data represent mean ± SD, **P < 0.01, ***P < 0.001 compared with HSF irradiated with 4 J/cm^2^ UVA group. **B** Normal HSF morphology (left) and HSF morphology after 16 J/cm^2^ UVA irradiation (right), scale bar = 200 μm, n = 4. **C** HSF cell viability after irradiation with different concentrations of resveratrol + 16 J/cm^2^ UVA, n = 4, data represent mean ± SD, **P < 0.01, ***P < 0.001 compared with 0 μM RSV group. **D** HSF morphology after irradiation with 16 J/cm^2^ UVA (left) and after irradiation with 100 μM RSV + 16 J/cm^2^ UVA HSF morphology (right), scale bar = 200 μm, n = 4. **E** Protein expression levels of Collagen I and MMP1 from different groups. **F–G** Relative expression levels of Collagen I and MMP1, n = 3. Data represent mean ± SD, **P < 0.01, ***P < 0.001 compared with Control group; ^*#*^*P* < 0.05, ^*##*^*P* < 0.01 compared with UVA group

UVA exposure leads to the photoaging of cells. In vitro models of photoaging were first induced using various UVA dosages. The results showed that UVA exposure could reduce the cell viability of HSF, and the cell viability gradually decreased with the increase of dose (Fig. 1A). At 16 J/cm^2^ UVA exposure, the cell viability was 54.11%. Normal HSF had a spindle-shaped morphology and good cell growth status, but UVA-irradiated HSF was severely atrophied, with irregular morphology and increased cellular debris (Fig. 1B), which indicated that we had successfully established a model of UVA-induced aging of HSF. To verify whether resveratrol could ameliorate photoaging caused by UVA exposure, we pretreated HSF with different concentrations of resveratrol ranging from 0 to 200 μM and administered 16 J/cm^2^ UVA exposure. The results showed that the cell viability was 81.17% when the resveratrol concentration was 100 μM, and 57.03% when the resveratrol concentration was 200 μM (Fig. 1C). Meanwhile, the pretreatment with resveratrol also reduced the cell morphology disruption caused by UVA, and the cell debris was significantly reduced (Fig. 1D). Resveratrol ameliorated the diminished viability and morphological disruption of HSF caused by UVA exposure. Furthermore, based on the results of the western blot (Fig. 1E–G), it was discovered that UVA significantly decreased collagen I expression and increased MMP1 expression. However, resveratrol treatment up-regulated collagen I and reduced MMP1 expression.

### Resveratrol promotes autophagy in HSF

**Fig. 2:**
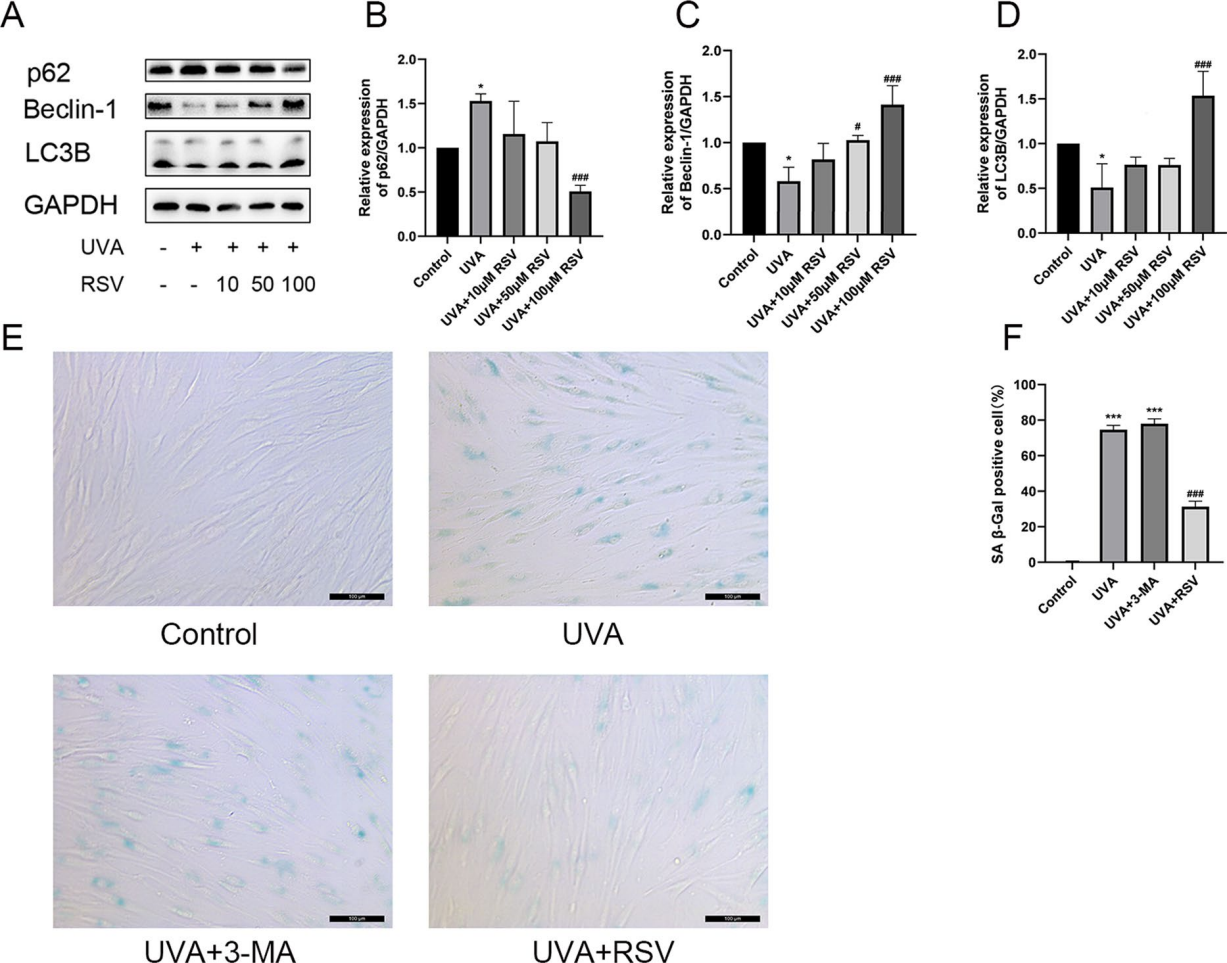
Resveratrol promotes autophagy in HSF. **A** Protein expression levels of p62, Beclin-1, and LC3B after pretreatment with different concentrations of resveratrol, n = 3. **B–D** Relative expression levels of p62, Beclin-1, and LC3B proteins, n = 3. **E** Results of β-galactosidase staining in different groups, scale bar = 100 μm, n = 4. **F** β-galactosidase activity levels in different groups, n = 4. Data represent mean ± SD, *P < 0.05, ***P < 0.001 compared with Control group; ^*#*^*P* < 0.05, ^*###*^*P* < 0.001 compared with UVA group

Since it has been established that cellular autophagy inhibition causes senescence (Gu et al. 2020), we examined the expression levels of the autophagy-related proteins p62, Beclin-1, and LC3B in HSF following UVA exposure before investigating the effects of pretreating HSF with various concentrations of resveratrol. The findings demonstrated that HSF levels of LC3B and beclin-1 expression were considerably reduced following UVA irradiation at a dosage of 16 J/cm^2^, but p62 protein expression was elevated (Fig. 2A–D). When compared to the UVA group, treatment with 100 μM resveratrol dramatically enhanced the expression of LC3B and Beclin-1 and decreased the expression of p62 (Fig. 2A–D). Similarly, it was also discovered that resveratrol alone increased the expression of the autophagy markers LC3B and Beclin-1 while lowering p62 expression (Fig. S1A–D). We pretreated HSF with 3-MA and resveratrol, gave them 16 J/cm^2^ of UVA exposure, and then watched for the SA-β-gal activity linked to senescence because research has shown that 3-MA can prevent autophagy (Bao et al. 2018). The outcomes showed that UVA irradiation greatly raised the SA-β-gal activity level of HSF. The SA-β-gal activity level was significantly lower following pretreatment with resveratrol (Fig. 2E, F), although it was higher in the group with the addition of the autophagy inhibitor 3-MA compared to the UVA group (Fig. 2E, F). According to the data discussed above, resveratrol can activate autophagy to slow down the aging process while suppression of autophagy can speed up the process.

### AMPK participates in autophagy to ameliorate UVA-induced photoaging

**Fig. 3:**
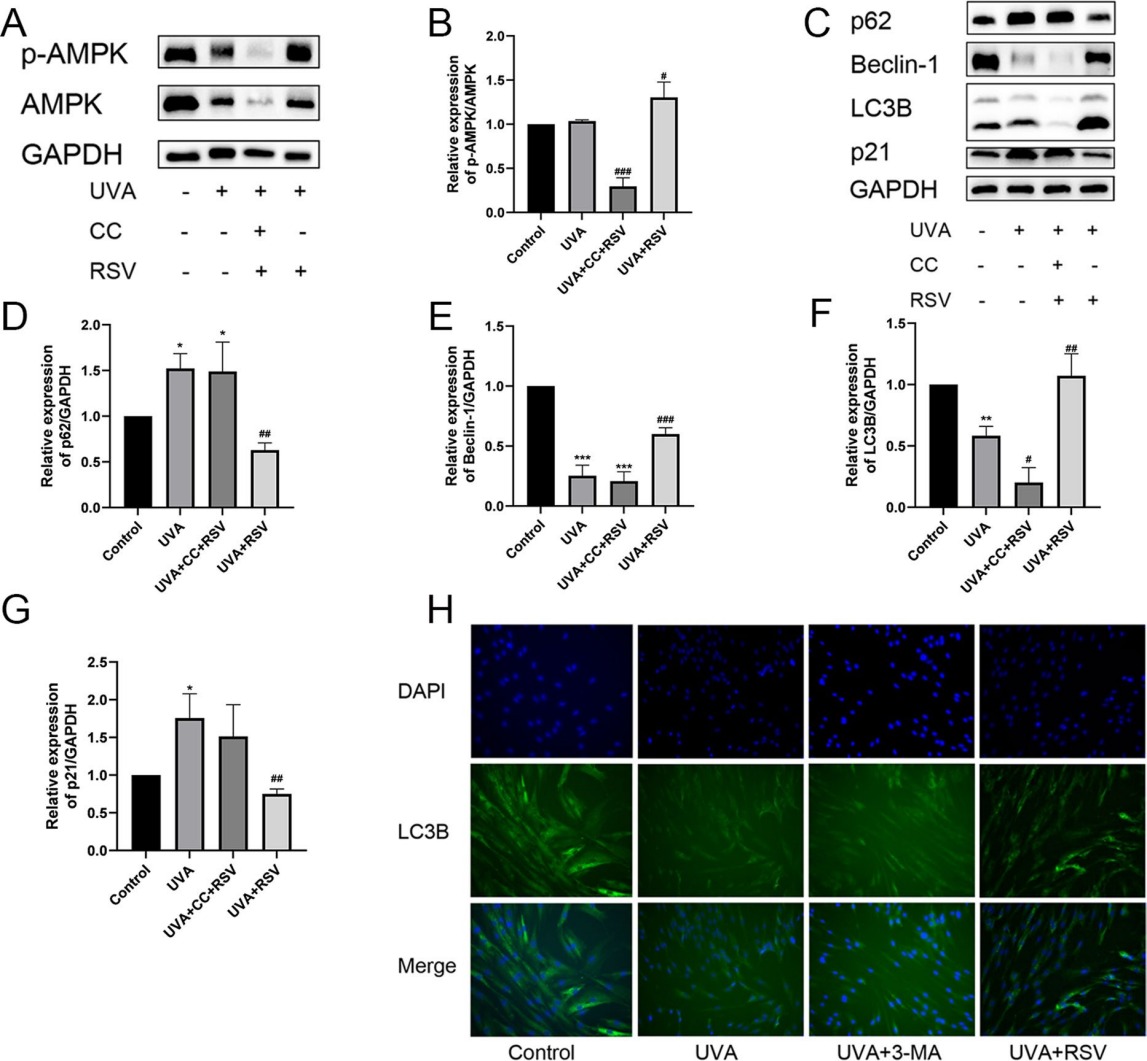
Resveratrol promotes autophagy through the AMPK signaling pathway. **A** HSF aging model was induced by 16 J/cm^2^ UVA, pre-treated with Compound C and 100 μmol/L RSV, and the expression levels of p-AMPK and AMPK were determined by Western blot. **B** Relative expression levels of p-AMPK/AMPK, n = 3. **C** Western blot of p62 in different groups, Beclin-1, LC3B, and p21 expression levels. **D–G** Relative expression levels of p62, Beclin-1, LC3B, and p21, n = 3. **H** Immunofluorescence staining results of LC3B in different groups, n = 5, scale bar = 100 μm. Data represent mean ± SD, *P < 0.05, **P < 0.01, ***P < 0.001 compared with Control group; ^*#*^*P* < 0.05, ^*##*^*P* < 0.01, ^*###*^*P* < 0.001 compared with UVA group

Although autophagy and the AMPK signaling route are closely connected (Mihaylova and Shaw 2011), it is unclear whether resveratrol promotes autophagy via the AMPK signaling pathway to reduce UVA-induced photodamage. After pretreatment with the AMPK inhibitor Compound C and 100 μmol/L RSV to look into the mechanism, we discovered that the expression levels of AMPK and p-AMPK were decreased after 16 J/cm^2^ UVA irradiation (Fig. 3A). Resveratrol and Compound C were combined to further lower their expression and p-AMPK/AMPK levels. In contrast, resveratrol boosted AMPK phosphorylation, which raised p-AMPK/AMPK expression levels (Fig. 3A, B, S1F, G). By using Western blot, we further investigated the expression levels of p62, Beclin-1, and LC3B. We discovered that exposure to 16 J/cm^2^ UVA boosted the expression of p62 while decreasing the expression of LC3B and Beclin-1 (Fig. 3C–G). Resveratrol and Compound C were administered together, which further reduced the expression of LC3B and Beclin-1 and markedly enhanced the expression of p62. The intracellular expression of LC3B and Beclin-1 was dramatically raised in the resveratrol group, whereas the expression of the protein p62 was significantly lowered (Fig. 3C, G, S1A–E). Additionally, we discovered that resveratrol dramatically decreased the expression of p21, but 16 J/cm^2^ UVA irradiation considerably elevated the expression of the senescence marker protein p21, which was further up-regulated in the co-treated group (Fig. 3C, G, S1A–E). By using immunofluorescence to further analyze the expression of LC3B in various treatment groups, we discovered that the amount of LC3B expression was increased following resveratrol treatment (Fig. 3H, S1H). The above results suggest that resveratrol can activate autophagy to ameliorate UVA-induced photoaging via the AMPK signaling pathway.

### Resveratrol promotes autophagy to reduce ROS production

**Fig. 4:**
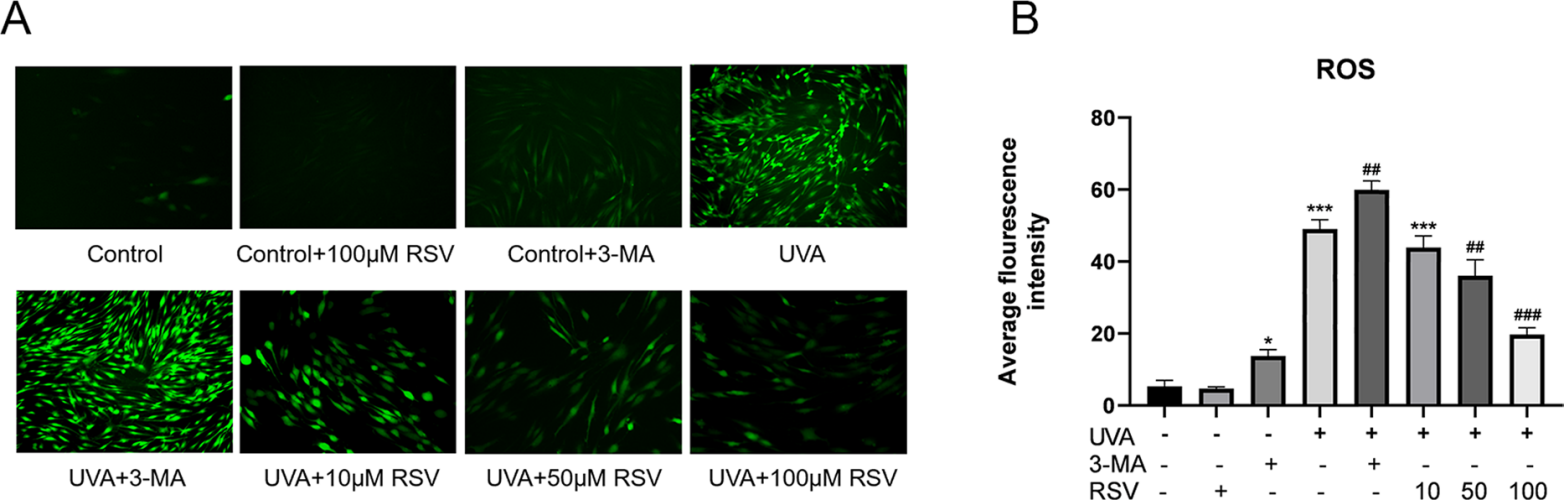
Resveratrol attenuates ROS levels induced by UVA irradiation. **A** ROS levels in HSF after different treatments, n = 3, scale bar = 100 μm. **B** Results of ROS fluorescence intensity analysis, n = 3. Data represent mean ± SD, *P < 0.05, ***P < 0.001 compared with the Control group; ^*##*^*P* < 0.01 and ^*###*^*P* < 0.001 compared with the UVA group

The main factor in photoaging is ROS, which is produced in considerable quantities by HSF after prolonged UVA exposure (Battie et al. 2014). As a result, we looked into the possibility that resveratrol could reduce ROS generation. HSFs were first treated with 3-MA and RSV alone, and then pretreated with 3-MA, 10 μmol/L RSV, 50 μmol/L RSV, and 100 μmol/L RSV before being exposed to 16 J/cm^2^ UVA radiation. When compared to the Control group, ROS levels were observed to have dramatically increased following UVA exposure, and the autophagy inhibitor 3-MA treatment only served to raise those levels (Fig. 4A, B). These findings show that ROS levels rise when autophagy is inhibited. There was a link between ROS levels and resveratrol concentration, and resveratrol therapy considerably reduced ROS levels (Fig. 4A, B). However, more research will be required to rule out the possibility that resveratrol’s inherent antioxidant properties are linked to the decrease in ROS.

### Effects of resveratrol on apoptosis and cycle in photoaging cells

**Fig. 5:**
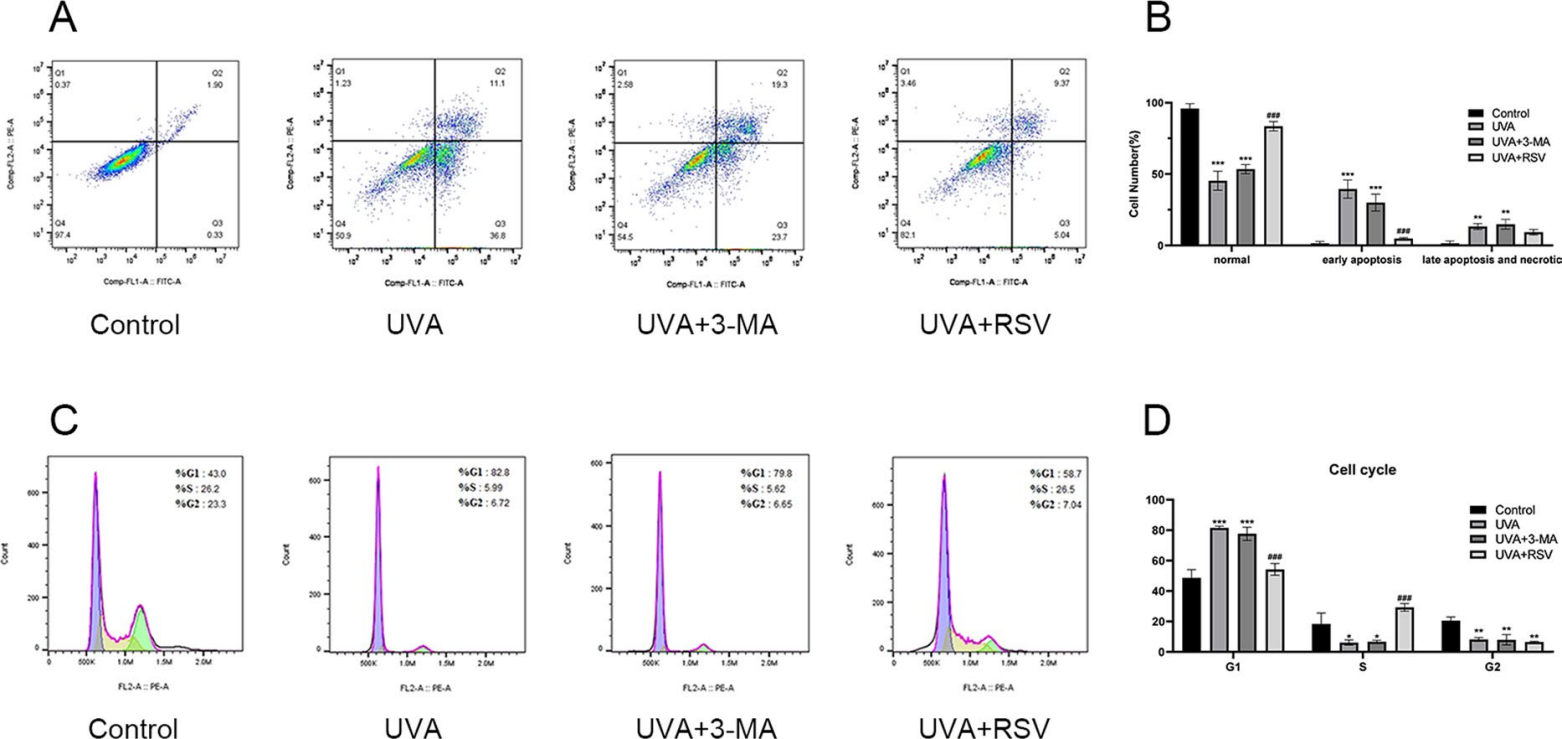
Effect of resveratrol on apoptosis and cycle of HSF photoaging. **A** Apoptosis of different constituent HSF. **B** Statistical results of normal, early apoptotic, late apoptotic, and necrotic cells in different groups, n ≥ 3. **C** Results of the cycle of different constituent HSF. **D** Statistical results of different constituent HSF in the G1, S, and G2 phases, n = 3. Data represent mean ± SD, *P < 0.05, **P < 0.01, ***P < 0.001 compared with the Control group; ^*###*^*P* < 0.001 compared with UVA group

After exposure to UVA radiation, cell morphology was disturbed and cellular debris increased. In order to examine how resveratrol affects apoptosis in UVA-induced photoaging, HSF was pretreated with 3-MA and 100 μmol/L RSV before being exposed to 16 J/cm^2^ UVA radiation. The findings demonstrated a considerable increase in apoptosis caused by UVA irradiation, with rates of early apoptosis rising from 0.33 to 36.8% and late apoptosis rising from 1.90 to 11.1% (Fig. 5A). Autophagy inhibition slightly increased apoptosis, however the 3-MA group further increased late apoptotic cells from 11.1 to 19.3% at the level of the UVA group (Fig. 5A). The amount of apoptosis was markedly reduced by resveratrol therapy, and the early apoptosis rate dropped to 5.04% (Fig. 5A, B). Since cell cycle arrest is another sign of cellular senescence (Ogrodnik et al. 2019), we investigated further how resveratrol affected the UVA-induced photoaging cell cycle. The findings demonstrated that UVA could significantly cause G1-phase arrest and that the G1-phase of UVA-irradiated fibroblasts increased from 43.0 to 82.8% (Fig. 5C). Restoring the number of cells entering the S-phase and G2-phase, resveratrol considerably reduced the UVA-induced G1-phase arrest to 58.7% (Fig. 5C, D).

### Resveratrol alleviates photoaging in mouse skin

**Fig. 6:**
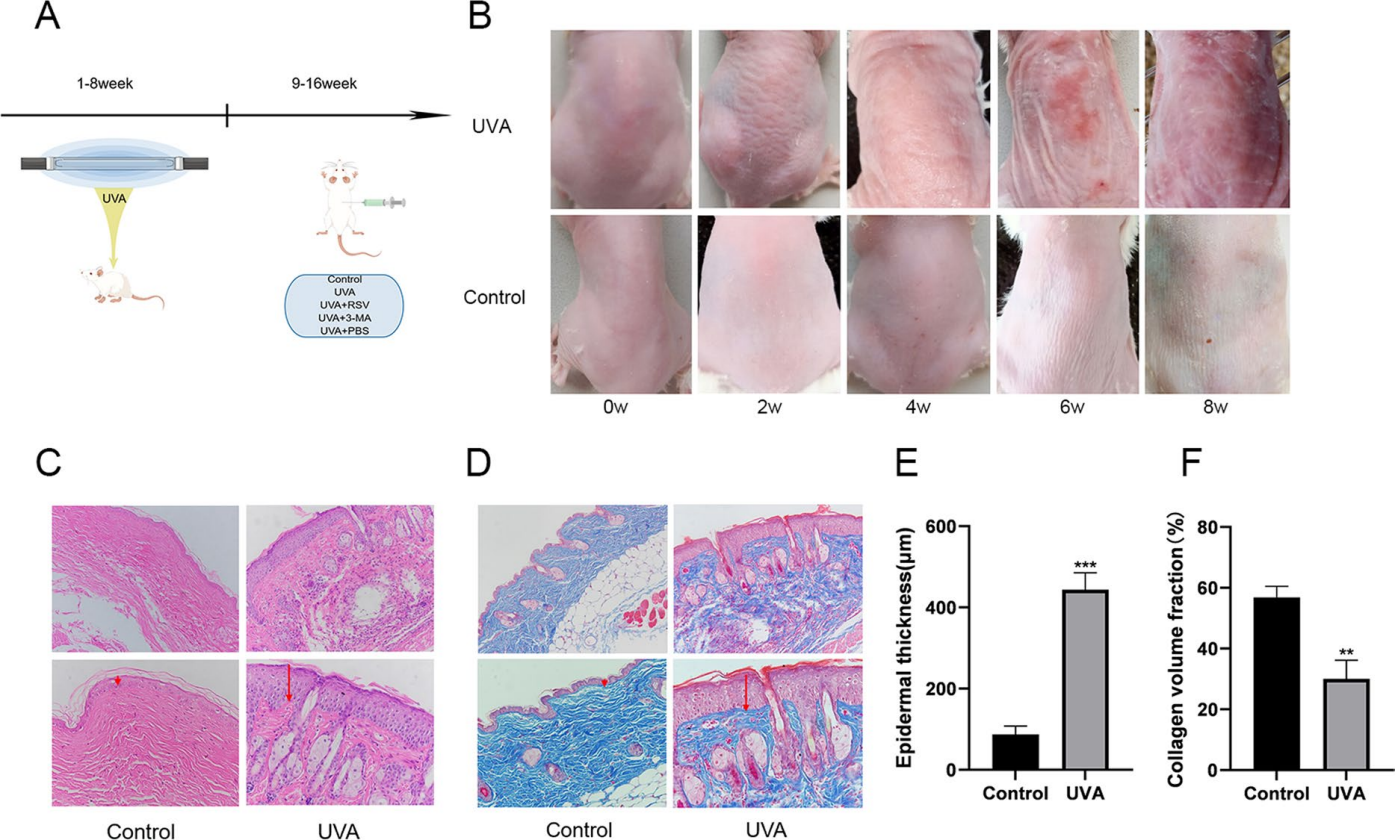
UVA can induce photoaging in mouse skin. **A** Schematic diagram of animal experiments (By Figdraw). **B** Appearance of dorsal skin of control and UVA-irradiated mice at 0–8 weeks. **C** HE staining results of control and UVA groups of mice, n = 3. **D** Masson staining results of control and UVA groups of mice, n = 3. **E** Statistical results of the thickness of the epidermal layer of mice in different groups, n = 3. **F** Collagen volumetric fractions of mice in different groups statistical results, n = 3. Data represent mean ± SD, **P < 0.01, and ***P < 0.001 compared with the Control group

The aforementioned findings demonstrated that resveratrol might reduce UVA-induced photoaging in fibroblasts, and we subsequently confirmed this effect in mice. To creatively induce photoaging in a mouse model, we combined 8-MOP with UVA (Fig. 6A). In comparison to the control group, the UVA group’s mice had skin that had more erythema, wrinkles, and hyperpigmentation after 8 weeks (Fig. 6B). HE staining and Masson staining also demonstrated that the control mice’s epidermis was well-organized, with a distinct epidermal-dermal demarcation, as well as that the dermis’ wavy fibrous tissues were uniformly distributed and arranged in an orderly fashion without any inflammatory cell infiltration (Fig. 6C, D). The mice in the UVA group had hyperplastic epidermis with hyperkeratosis (Fig. 6E); on the contrary, the dermis’ collagen fibers had degraded, been damaged, and decreased; they were also disorganized and unevenly distributed, and there had been an increase in inflammatory cell infiltration (Fig. 6F). The aforementioned findings suggested that the UVA-induced photoaging mouse model was successfully recreated.

**Fig. 7:**
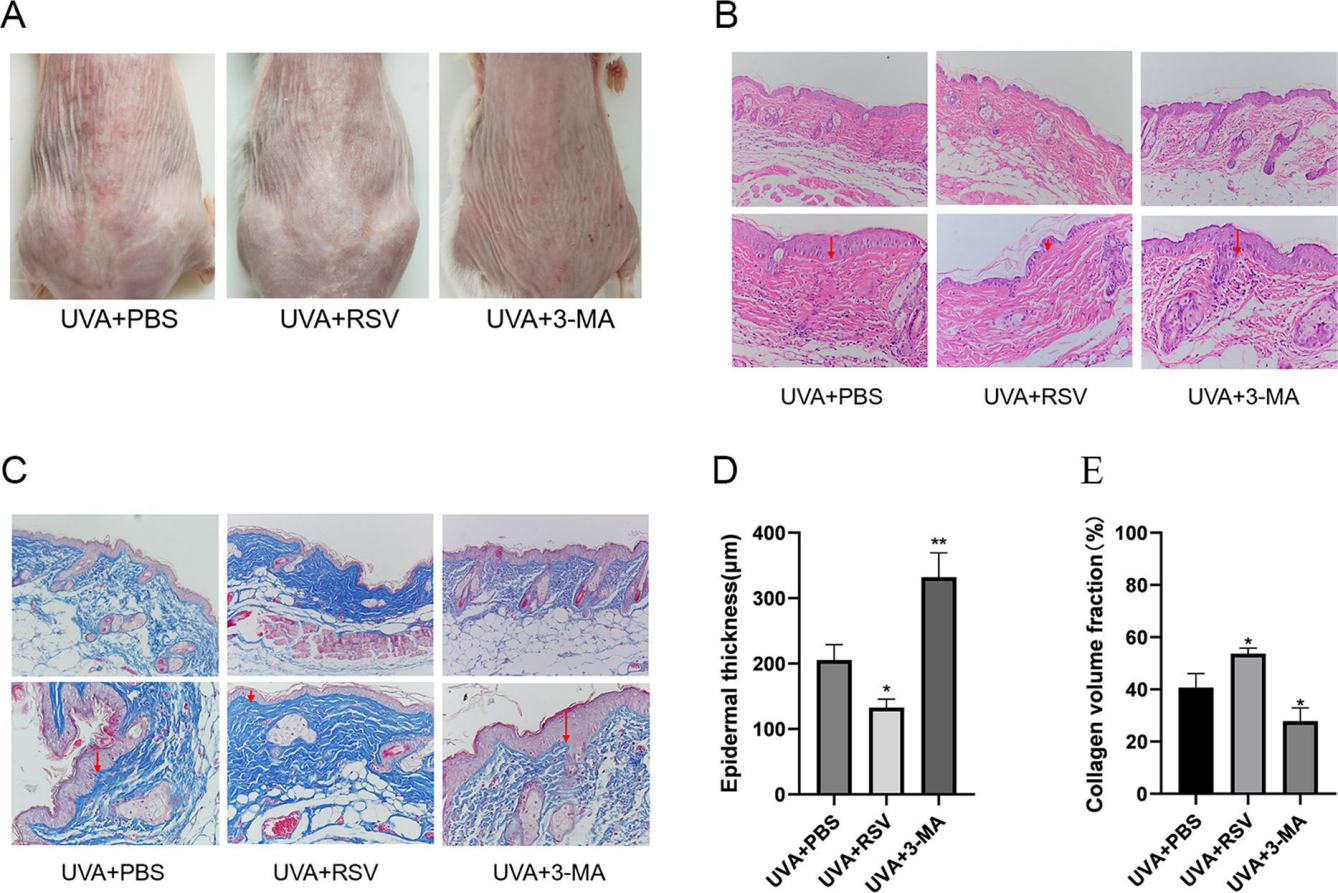
Resveratrol alleviates photoaging in mouse skin. **A** Appearance of dorsal skin of photoaged mice after subcutaneous injection of PBS, RSV, and 3-MA. **B** Results of HE staining of dorsal skin of mice in different groups, scale bar = 100 μm. **C** Results of Masson staining of dorsal skin of mice in different groups, scale bar = 100 μm. **D** Resulting in statistical plots of the epidermal thickness of mice in different groups, n = 3. **E** Collagen volume of mice in different groups Statistical graphs of the results of fractions, n = 3. Data represent mean ± SD, *P < 0.05, and **P < 0.01 compared with the UVA + PBS group

We injected PBS, RSV, and 3-MA into the dorsal skin of photoaged mice in order to investigate the effects of resveratrol on these animals. We found that resveratrol improved photoaging phenomena, such as increased erythema and wrinkles in the mice’s dorsal skin, and that 3-MA worsened aging phenomena when compared to the UVA + PBS group (Fig. 7A). By using HE staining and Masson staining, it was possible to determine that the mice in the UVA + 3-MA group had much less epidermal thickening and collagen fibers than the mice in the UVA + PBS group, which was accompanied by a significant infiltration of inflammatory cells (Fig. 7B–E). After receiving resveratrol treatment, there was a considerable reduction in the infiltration of inflammatory cells and the necrotic shedding of epidermal cells (Fig. 7B, C). In mice, subcutaneous injection of resveratrol dramatically reduced hyperkeratosis, decreased epidermal layer thickness, and increased collagen fiber content (Fig. 7D, E). Together, our findings showed that resveratrol dramatically reduced UVA-induced photoaging in mouse skin and that suppression of autophagy increased photoaging.

### Resveratrol ameliorates skin photoaging in mice via AMPK-activated autophagy

**Fig. 8:**
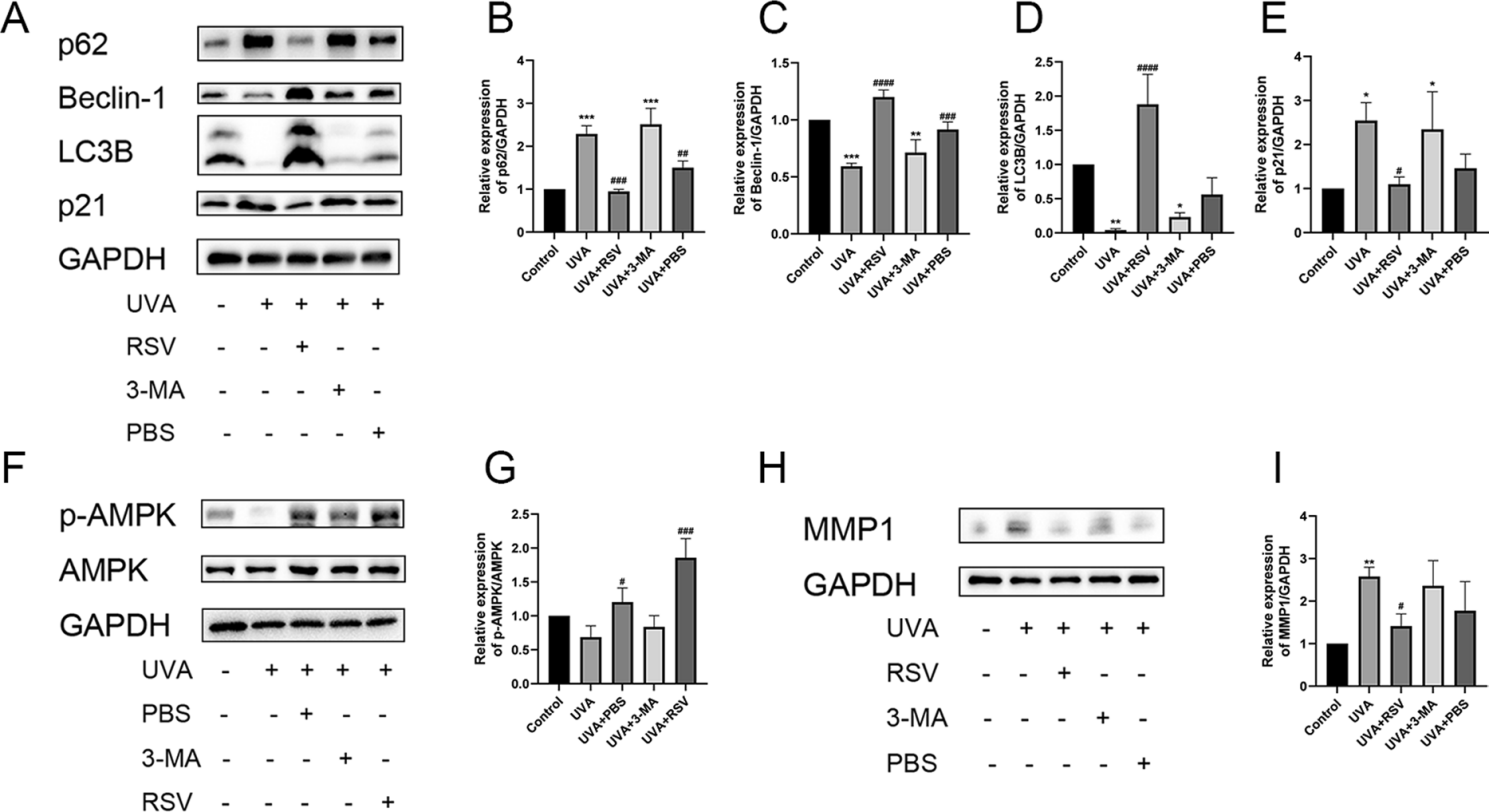
Resveratrol ameliorates skin photoaging in mice through AMPK-activated autophagy. **A** Protein expression levels of p62, Beclin-1, LC3B, and p21 in dorsal skin tissues of mice from different groups. **B–E** Relative expression levels of p62, Beclin-1, LC3B and p21, n = 3. **F** Protein expression levels of p-AMPK and AMPK in dorsal skin tissues of mice from different groups. **G** p-AMPK and AMPK relative expression levels, n = 3. **H** Protein expression levels of MMP1 in the dorsal skin tissues of mice in different groups. **I** Relative expression levels of MMP1, n = 3. Data represent mean ± SD, *P < 0.05, **P < 0.01, ***P < 0.001 compared with Control group; ^*#*^*P* < 0.05, ^*##*^*P* < 0.01, ^*###*^*P* < 0.001, ^*####*^*P* < 0.0001 compared with UVA group

We performed protein immunoblotting tests to determine if resveratrol improved photoaging mice connected to autophagy, as shown by the data above that showed autophagy inhibition aggravated photoaging and resveratrol improved photoaging. According to the findings, UVA irradiation considerably reduced the expression of LC3B and Beclin-1 in the back skin tissues of photoaged mice compared to the control group while dramatically increasing the expression levels of p62 and p21 proteins (Fig. 8A–E). The expression of LC3B and Beclin-1 was considerably greater and the expression of p62 and p21 were significantly decreased in the resveratrol-treated group respectively (Fig. 8A–E). We also discovered that p-AMPK/AMPK expression was dramatically downregulated by UVA, but p-AMPK/AMPK expression was significantly upregulated by resveratrol (Fig. 8F, G). Because MMP1 is essential for photoaging and controls the breakdown of type I and type III collagen (Pittayapruek et al. 2016) we also looked at the level of MMP1 protein expression in the dorsal skin tissues of photoaged mice. The outcomes demonstrated that while resveratrol therapy decreased MMP1 expression, UVA irradiation significantly increased MMP1 expression (Fig. 8H, I). The above results demonstrated that resveratrol may activate autophagy via AMPK to improve skin photoaging in mice.

## Discussion

Long-term UV radiation exposure promotes skin photoaging (Gilchrest 2013), which alters the skin’s tissue and functional properties. Prolonged exposure to UVA radiation impairs autophagy (Ma et al. 2022), produces copious ROS, and activates signaling pathways such as Nuclear factor-κB (NF-κB) (Amaro-Ortiz et al. 2014; Chen et al. 2021), upregulates the production of Tumor necrosis factor-α (TNF-α) and MMP (Salminen et al. 2022), alters the structure of the extracellular matrix (Amano 2016; Rabe et al. 2006; Rittie and Fisher 2015)and results in DNA damage (Brem et al. 2017; Sage et al. 2012). In this study, we used resveratrol to modify a UVA-induced photoaging model in order to examine the anti-aging mechanism of resveratrol. The findings demonstrated that various UVA dosages decreased cell viability and altered cell shape. By phosphorylating AMPK and inducing autophagy, resveratrol can upregulate the expression of LC3B, and Beclin-1, and decrease the levels of p62 and p21. Resveratrol additionally decreased UVA-induced G1 cell cycle arrest and prevented ROS generation and apoptosis in the photoaging model, which mitigated UVA-induced photoaging. Furthermore, by combining 8-MOP and UVA, we were able to induce photoaging in a mouse model. Resveratrol administered subcutaneously dramatically reduced mouse back erythema, wrinkles, and deep folds. Resveratrol reduced the signs of hyperkeratosis, reduced the thickness of the epidermal layer, and increased the amount of collagen fibers in mouse skin’s epidermal layer. In conclusion, our cell and animal studies showed that resveratrol can reduce UVA-induced photoaging by activating the AMPK signaling pathway.

A previous study found that resveratrol protects human keratinocytes (HaCaT) from UVA-induced oxidative stress damage by downregulating Keap1 expression (Liu et al. 2011). Zhou et al. found that resveratrol protected HaCaT cells from UVB-induced photoaging by upregulating HSP27 and modulating the mitochondrial cysteine-dependent apoptotic pathway (Zhou et al. 2018). Although the aforementioned findings showed that resveratrol may have a function in the therapy of photoaging, most studies only looked at the cellular level and the impact of resveratrol on the skin of photoaged mice has not been documented. In our study, we proved that resveratrol has a protective effect against UVA-induced photoaging from both mouse and cellular perspectives, and optimized the anti-aging mechanism of resveratrol.

In photoaging mice, modeling typically lasts 10 weeks or longer (LH, 1989 Sep; Liang et al. 2007) and high-dose UV irradiation frequently results in acute photodamage in mice, which is also inconsistent with actual UV exposure in humans. As a result, we built an innovative, more clinically applicable method of modeling on the foundation of the earlier approach. We began irradiation with a minimum dose of 0.1 J/cm^2^ to prevent acute photodamage in mice. We calculated the lowest dose of 0.35 J/cm^2^, which caused skin erythema, and then increased the dose by 3–5% from the dose from the day before. If the mice showed erythema or inflammatory damage on the 2nd day of exposure, they were rested for 2 days, and thereafter the previous dose was continued, with a 1-day pause after four consecutive days of exposure. The successful mouse photoaging model was created in under 8 weeks. This modeling approach offers a fresh modeling concept for preclinical research of photoaging while successfully avoiding acute photodamage brought on by UV irradiation, and being more applicable to preclinical studies of photoaging. Additionally, we opted to inject the medication subcutaneously into the mice’s back wrinkles rather than topical application, which facilitates the drug’s absorption and action in the dermis.

ROS are a byproduct of oxidation on all biomolecules (lipids, proteins, nucleic acids, and carbohydrates) in a series of oxidative processes, and they have been related to the emergence of several diseases (Forrester et al. 2018). Furthermore, ROS causes cellular senescence because it impairs DNA transcription and mRNA translation, obliterates protein structure and function, and lowers cellular activity and metabolic capacity. By releasing inflammatory molecules and disrupting mitochondrial homeostasis, senescent cells impair the immunological and metabolic activities of normal cells. This creates a vicious cycle where more ROS are produced, which in turn encourages cellular senescence. This study furthered the significance of resveratrol in anti-aging by demonstrating that it can reduce ROS generation via encouraging autophagy.

While our findings observed an association between resveratrol treatment and increased autophagic activity mediated through AMPK signaling, the exact mechanistic details were not exhaustively explored. According to research, resveratrol can either directly interact with upstream regulators of the AMPK pathway or modulate the state of cellular energy to indirectly activate AMPK(Ungurianu et al. 2023). Resveratrol suppresses the molecular target of mTOR (a negative autophagy regulator) when it activates AMPK(Sanches-Silva et al. 2020). This inhibition promotes the start of autophagy by reducing the limiting impact of mTOR on the ULK1 complex (Iorio et al. 2021). Additionally, ULK1 phosphorylation can be directly increased by AMPK activation (Iorio et al. 2021; Xiang et al. 2020). Moreover, it has been linked to the activation of Beclin-1, which aids in the creation of autophagosomes (Xu and Wan 2023). The process of AMPK signaling-mediated autophagy activation entails a series of phosphorylation events that culminate in critical autophagy machinery components, such as mTOR, Beclin-1, and ULK1(Salminen and Kaarniranta 2012). Subsequent research endeavors may explore additional signaling pathways implicated in skin aging processes, clarifying the ensuing direct connections and downstream consequences. Additionally, it is important to take into account resveratrol’s possible antioxidant and UV-blocking qualities, which may also be one of the ways it reduces photoaging. In addition, more research is necessary to translate our findings into clinical practice. Future research on the topical application of resveratrol on skin aging is necessary to gain insight into benefits that are specific to a given area. Finally, optimizing the dosage and frequency of administration may maximize its effectiveness and minimize any possible negative effects, which will be of great help for future clinical applications.

In conclusion, our innovative UVA-induced photoaging model in mice and in vivo confirmed the critical role of resveratrol in photoaging prevention. The prospective targets for the creation of novel clinical medications are provided by the putative molecular mechanisms of resveratrol for preventing photoaging. With the depletion of the ozone layer and the increased severity of UV-induced photoaging, we are going to conduct more comprehensive studies on active medications to reduce photoaging in the future to provide additional options for anti-aging. Furthermore, our research confirms that prolonged UVA exposure-induced autophagy inhibition speeds up the aging process in both mice and fibroblasts. Numerous other studies have also demonstrated that autophagy inhibition promotes aging. Consequently, at the level of molecules to the whole body and population, the degree of autophagy may be one of the factors determining the heterogeneity of the rate and extent of age-related changes at various levels of biological organization. It is anticipated that a growing number of thorough studies will undoubtedly be carried out in the future to confirm this conjecture.

### Supplementary Information

Below is the link to the electronic supplementary material.

Supplementary file 1 (PDF 200 KB)

## Data Availability

All data generated or analyzed during this study are available from the corresponding author on reasonable request.

## Abbreviations

UVA: Ultraviolet A
HSF: Human skin fibroblasts
AMPK: Adenosine monophosphate-activated protein kinase
p-AMPK: Phosphorylated adenosine monophosphate-activated protein kinase
ROS: Reactive oxygen species
ECM: Extracellular matrix
MMP: Matrix metalloproteinase
ULK1: Unc-51 like autophagy activating kinase 1
mTOR: Mechanistic target of rapamycin
RSV: Resveratrol
3-MA: 3-Methyladenine
CC: Compound C
8-MOP: 8-Methoxy psoralen
SA-β-gal: Senescence-associated β-galactosidase
MAPK: Mitogen-activated protein kinase
NF-κB: Nuclear factor-κB signaling pathways
TNF-α: Tumor necrosis factor-α
HaCaT: Human keratinocytes
