# Supplementary material for: Resveratrol activates autophagy and protects from UVA-induced photoaging in human skin fibroblasts and the skin of male mice by regulating the AMPK pathway
Source: Biogerontology. 2024 Apr 9;25(4):649–64. doi: 10.1007/s10522-024-10099-6 (PMC11217112; doi:10.1007/s10522-024-10099-6)
Supplement: Supplementary file 1 — (PDF 200 KB) [file 10522_2024_10099_MOESM1_ESM.pdf]

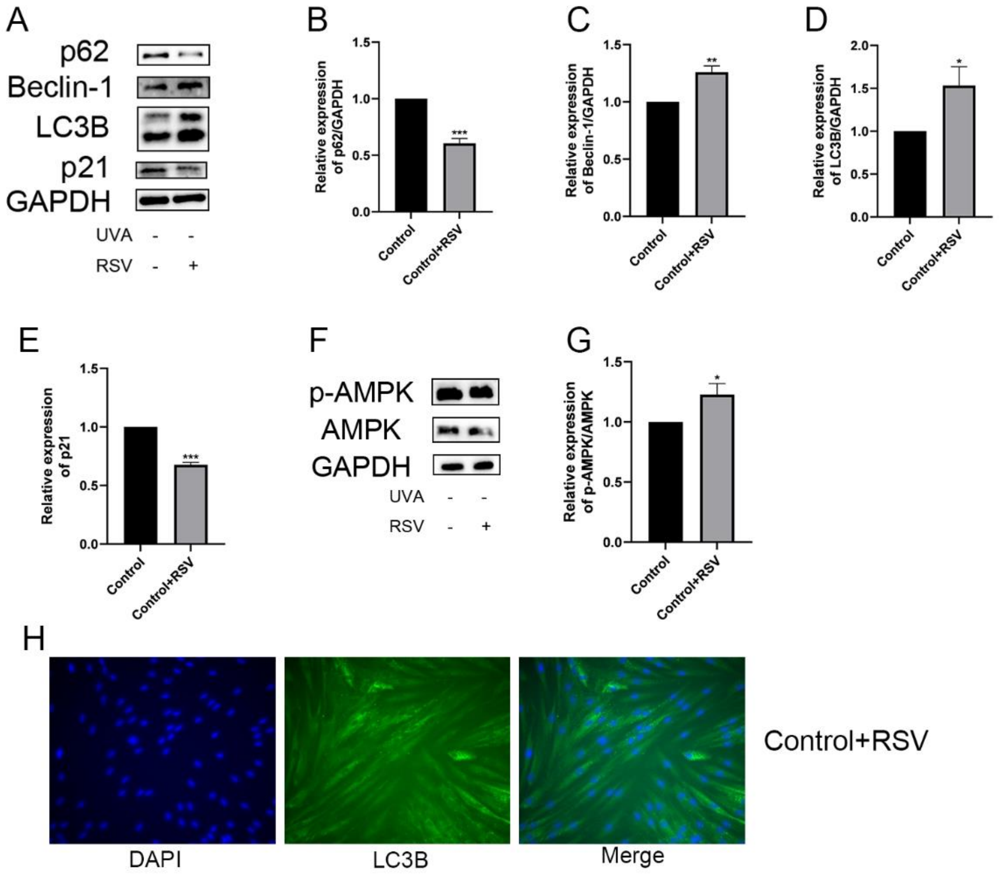

Fig.S1. Resveratrol promotes autophagy through the AMPK signaling pathway. (A) Western blot of p62, Beclin-1, LC3B, and p21 expression levels in the control group and resveratrol-alone group. (B-E) Relative expression levels of p62, Beclin-1, LC3B, and p21, n = 3. (F) The expression levels of p-AMPK and AMPK were determined by Western blot. (G) Relative expression levels of p-AMPK/AMPK, n = 3. (H) Immunofluorescence staining results of LC3B in the resveratrol-alone group, n = 3, scale bar = 100 μm. Data represent mean ± SD, $^{*}P < 0.05$, $^{**}P < 0.01$, $^{***}P < 0.001$ compared with the Control group.
